# Supplementary material for: Recurrent cancer‐associated ERBB4 mutations are transforming and confer resistance to targeted therapies
Source: Mol Oncol. 2025 Dec 23;20(5):1323–46. doi: 10.1002/1878-0261.70189 (PMC13155151; doi:10.1002/1878-0261.70189)
Supplement: Supplementary file 1 — Fig. S1. ERBB4 alterations in clinical cancer samples. Fig. S2. Biochemical activity and expression of ERBB4 variants in MCF10a cells in the absence of transformation pressure. Fig. S3. Role of ERBB3 in ERBB4‐mediated transformation of Ba/F3 cells. Fig. S4. Effect of prolonged serum starvation on ERBB4 wild‐type and S303F mutant activity in COS7 cells. Fig. S5. Tumor characteristics of patients harboring somatic transforming ERBB4 mutations. Table S1. Expression plasmids generated in this work. Table S2. Patients harboring ERBB4 alterations and treated with neratinib in the SUMMIT trial. [file MOL2-20-1323-s001.pdf]

SUPPORTING INFORMATION

Recurrent cancer-associated ERBB4 mutations are transforming and confer resistance to targeted therapies

Veera K. Ojala, Sini Ahonen, Sara Peltola, Aura Tuohisto-Kokko, Olaya Esparta, Peppi Suominen, Anne Jokilammi, Iman Farahani, Deepankar Chakroborty, Nikol Dibus, Steffen Boettcher, Tomi T. Airenne, Mark S. Johnson, Lisa D. Eli, Klaus Elenius, and Kari J. Kurppa

Supplementary Figure S1. ERBB4 alterations in clinical cancer samples.

Supplementary Figure S2. Biochemical activity and expression of ERBB4 variants in MCF10a cells in the absence of transformation pressure.

Supplementary Figure S3. Role of ERBB3 in ERBB4-mediated transformation of Ba/F3 cells.

Supplementary Figure S4. Effect of prolonged serum starvation on ERBB4 wild-type and S303F mutant activity in COS7 cells.

Supplementary Figure S5. Tumor characteristics of patients harboring somatic transforming ERBB4 mutations.

Supplementary Table S1. Expression plasmids generated in this work.

Supplementary Table S2. Patients harboring *ERBB4* alterations and treated with neratinib in the SUMMIT trial.

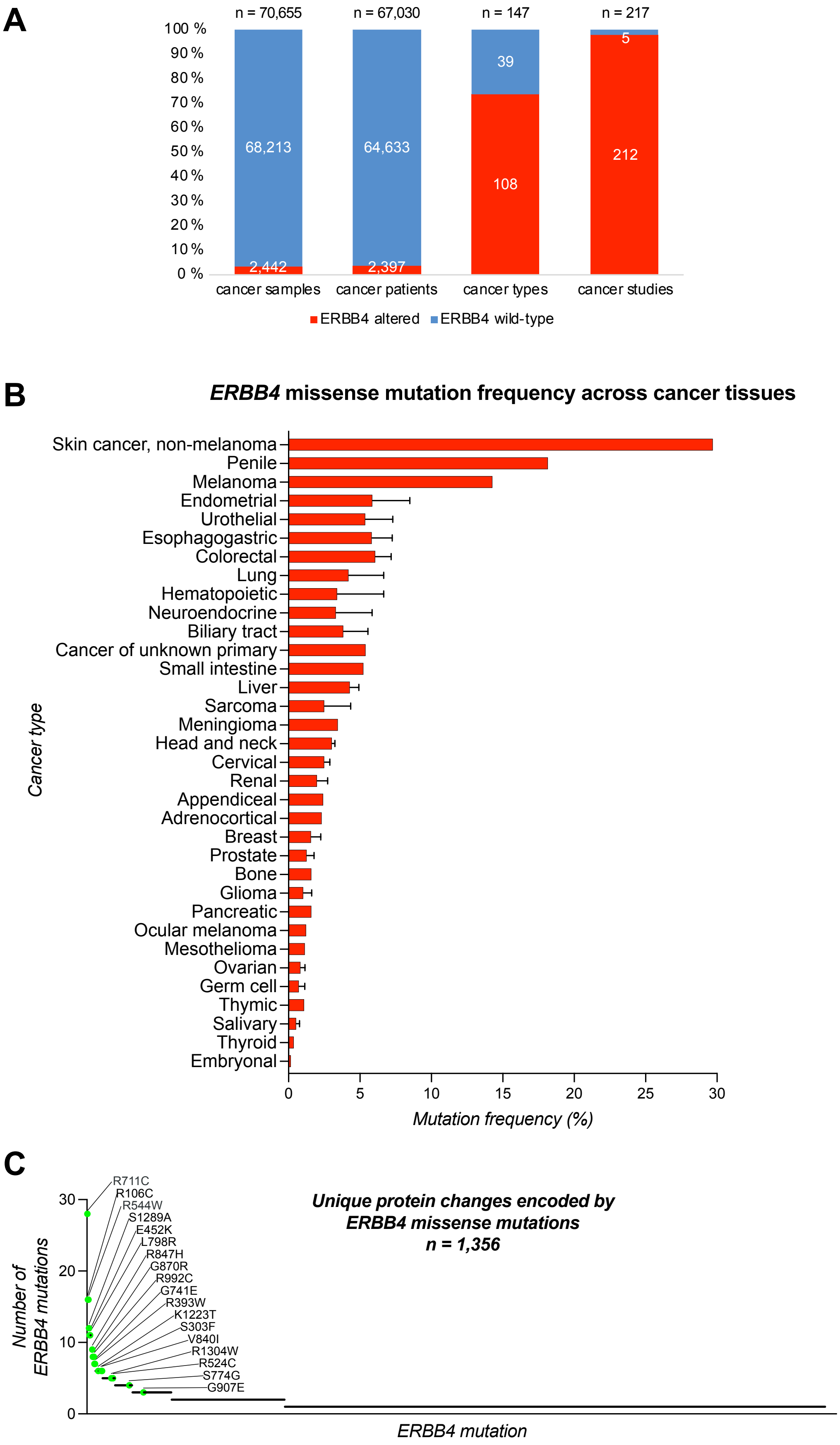
A
n = 70,655
n = 67,030
n = 147
n = 217
100 %
90 %
80 %
70 %
60 %
50 %
40 %
30 %
20 %
10 %
0 %
68,213
2,442
64,633
2,397
39
108
5
212
cancer samples
cancer patients
cancer types
cancer studies
ERBB4 altered
ERBB4 wild-type
B
ERBB4 missense mutation frequency across cancer tissues
Cancer type
Skin cancer, non-melanoma
Penile
Melanoma
Endometrial
Urothelial
Esophagogastric
Colorectal
Lung
Hematopoietic
Neuroendocrine
Biliary tract
Cancer of unknown primary
Small intestine
Liver
Sarcoma
Meningioma
Head and neck
Cervical
Renal
Appendiceal
Adrenocortical
Breast
Prostate
Bone
Glioma
Pancreatic
Ocular melanoma
Mesothelioma
Ovarian
Germ cell
Thymic
Salivary
Thyroid
Embryonal
0
5
10
15
20
25
30
Mutation frequency (%)
C
Unique protein changes encoded by
ERBB4 missense mutations
n = 1,356
Number of ERBB4 mutations
30
20
10
0
R711C
R106C
R544W
S1289A
E452K
L798R
R847H
G870R
R992C
G741E
R393W
K1223T
S303F
V840I
R1304W
R524C
S774G
G907E
ERBB4 mutation

Supplementary Figure S1. ***ERBB4*** **alterations in clinical cancer samples. A)** *ERBB4* alterations in clinical cancer samples reported in cBioPortal (https://cbioportal.org) curated non-redundant studies (January 2024). **B)** *ERBB4* missense mutation frequencies across cancer types reported in cBioPortal, classified by tissue of origin (mean with range shown). **C)** *ERBB4* missense mutations listed in cBioPortal, ranked by recurrence. The 18 recurrent mutations chosen for functional analyses are indicated.

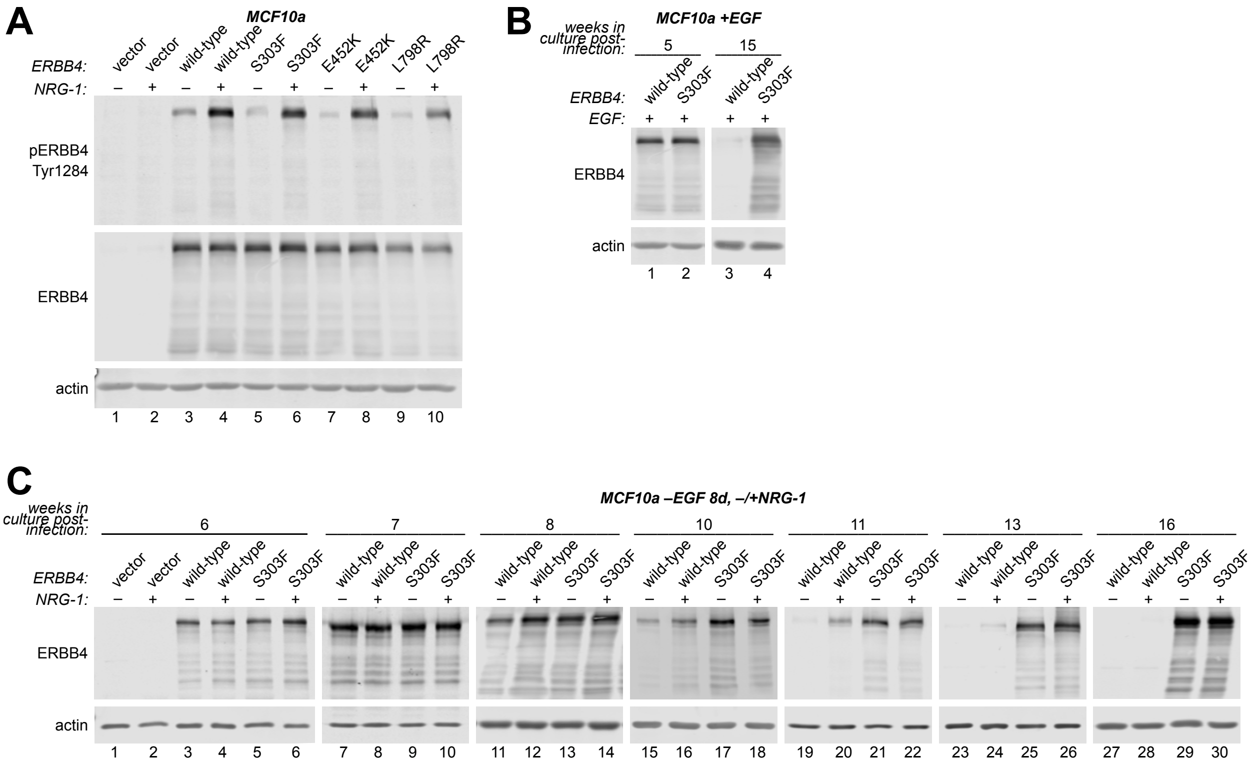

Supplementary Figure S2. Biochemical activity and expression of ERBB4 variants in MCF10a cells in the absence of transformation pressure. A) MCF10a cells stably expressing ERBB4 variants or vector control cells were serum starved overnight and stimulated or not with 50 ng/ml NRG-1 for 10 minutes. Cells were analyzed by western blot using beta-actin as a loading control. **B)** MCF10a cells stably expressing wild-type ERBB4 or the S303F variant were maintained in fully supplemented growth medium (including 20 ng/ml EGF per manufacturer's instructions), and in the presence of puromycin selection for indicated time after lentiviral infection of the cells with the ERBB4 encoding constructs. Cells were analyzed as in A. **C)** MCF10a cells stably expressing wild-type ERBB4, the S303F variant or vector control were subjected to eight-

day EGF deprivation in the presence or absence of 50 ng/ml NRG-1 (10% serum) after an indicated time in culture after lentiviral infection. Cell lysates were analyzed as in A. The lanes 19-22 are from the same experiment as Figure 4A.

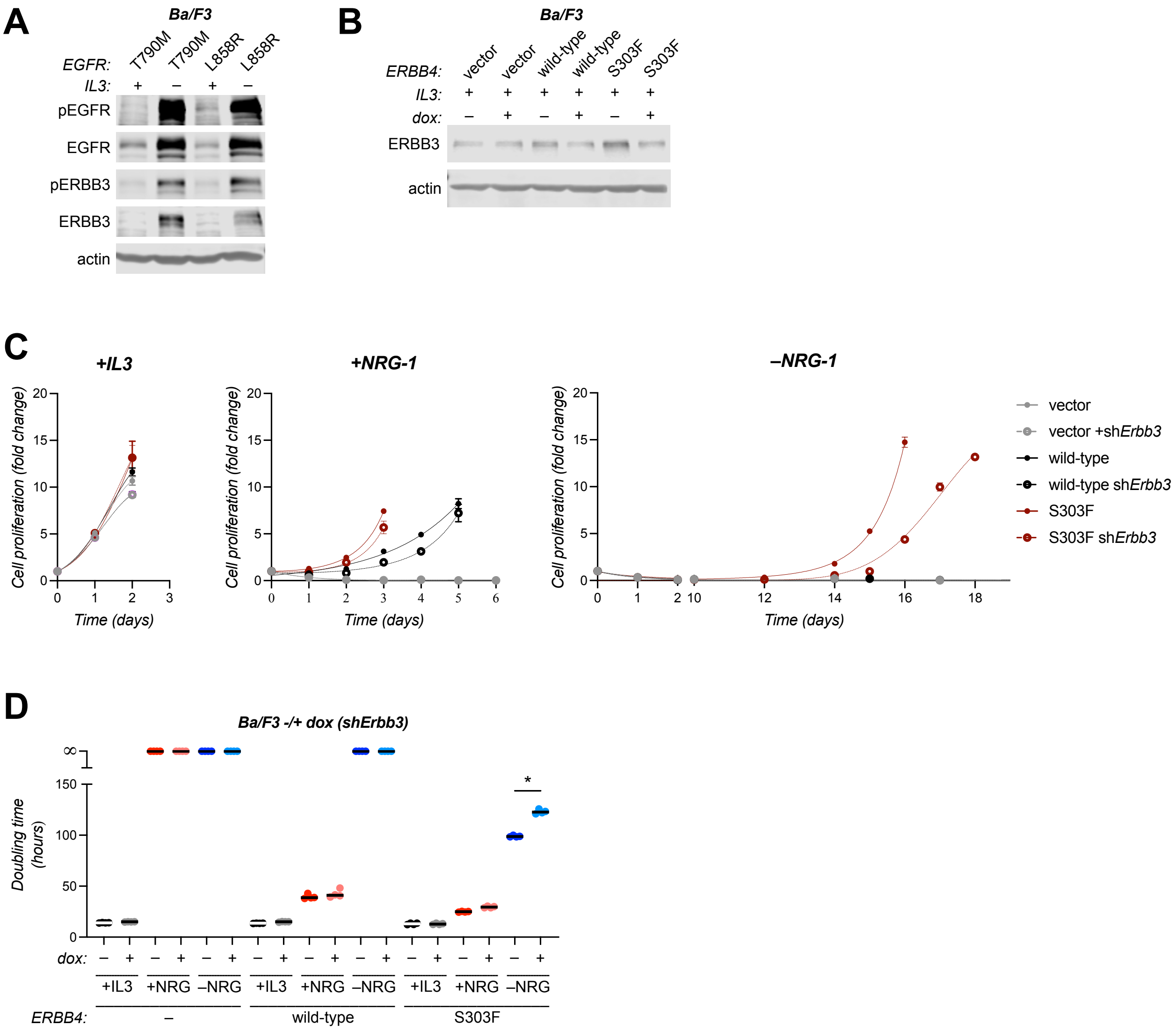

Supplementary Figure S3. Role of ERBB3 in ERBB4-mediated transformation of Ba/F3 cells. A) Ba/F3 cells stably expressing EGFR mutants were cultured in the absence or presence of IL3 and analyzed by western. **B)** Ba/F3 cells stably expressing ERBB4 variants or vector control together with doxycycline (dox)-inducible *Erbb3* shRNA were cultured in the absence or presence of dox and knockdown efficiency was analyzed by western using beta-actin as a loading control. **C)** Ba/F3 cells analyzed in B were cultured in the presence of IL3 or 20 ng/ml NRG-1 or in the absence of both, with or without dox (10% serum). Cell proliferation was measured with MTT

assay. **D)** Doubling times of ERBB4 variant-expressing Ba/F3 cells upon shRNA-mediated *Erbb3* knockdown. Welch two-sample *t* test was used for pairwise comparisons between -/+ dox samples from each IL3-independently growing cell lines expressing ERBB4 variants. P-values were corrected for multiple comparisons by Bonferroni method. *, $P < 0.0001$.

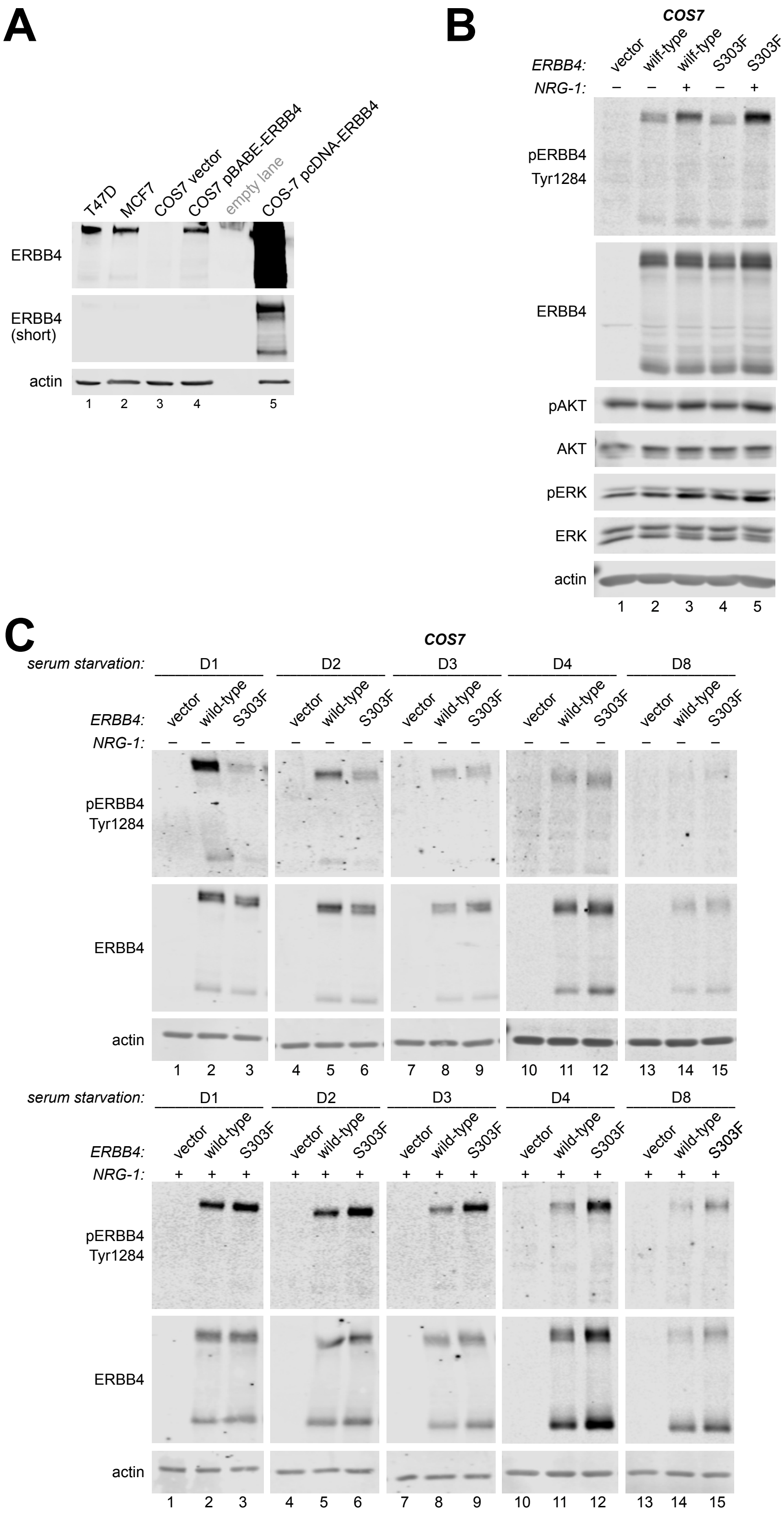

Supplementary Figure S4. Effect of prolonged serum starvation on ERBB4 wild-type and S303F mutant activity in COS7 cells. A) The endogenous expression levels of ERBB4 in T47D

and MCF7 cells, as well as overexpression levels in COS7 cells transiently transfected with equal amounts of *pBABE-puro-gateway-ERBB4JM-aCYT-2* plasmid, or *pcDNA3.1.-ERBB4JM-aCYT-2 plasmid*. **B**) COS7 cells were transfected with constructs encoding ERBB4 variants or vector control, then subjected to overnight serum starvation the next day and stimulated or not with 50 ng/ml NRG-1 for 10 minutes. Lysates were analyzed by western blot and loading was controlled with anti-actin. **C)** COS7 cells were transfected with constructs encoding ERBB4 variants or vector control. Next day, the cells were subjected to serum starvation in the presence or absence of 50 ng/ml NRG-1 for 1, 2, 3, 4 or 8 days, and lysates were analyzed as in B.

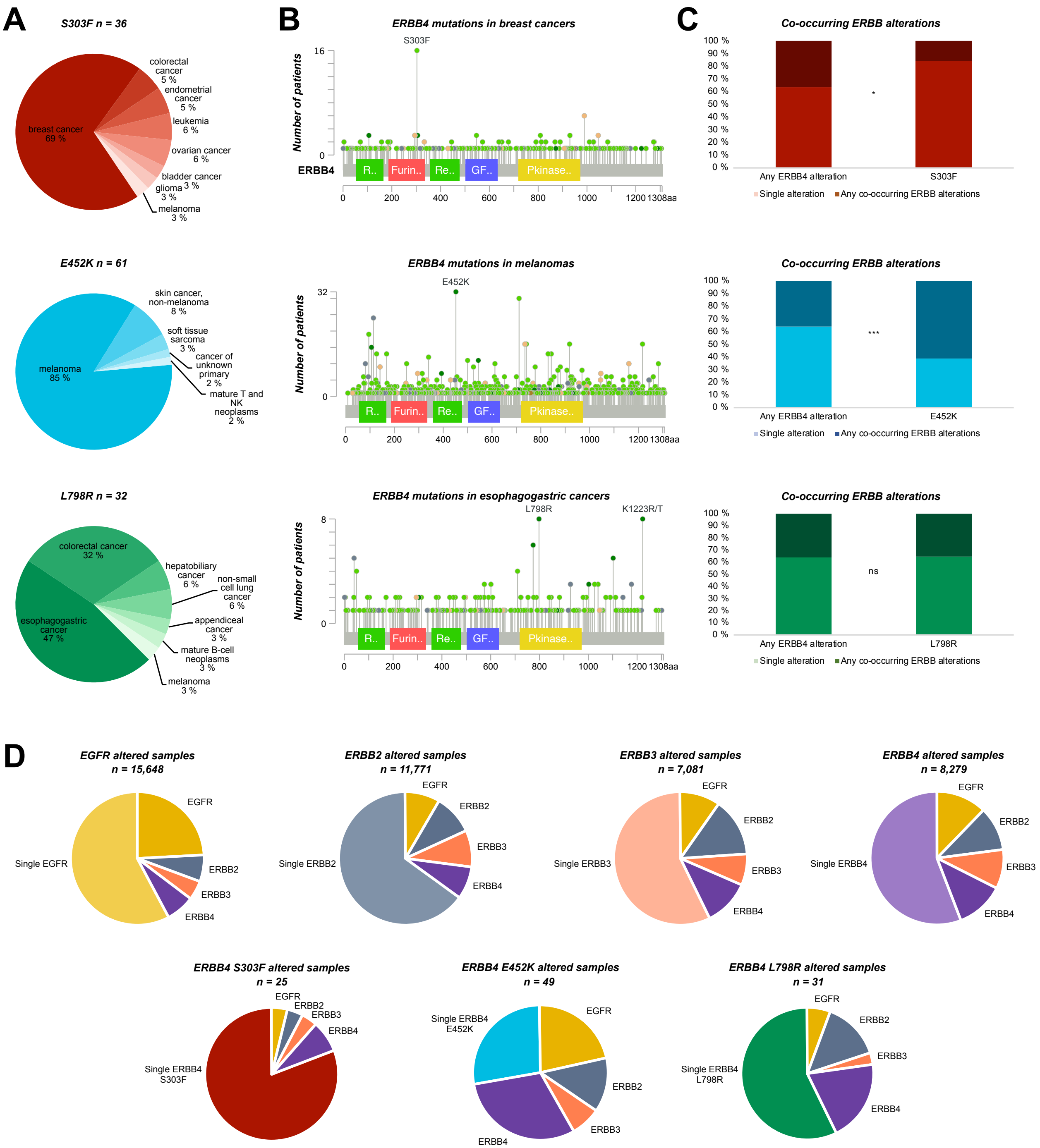

Supplementary Figure S5. Tumor characteristics of patients harboring somatic transforming ERBB4 mutations. A) ERBB4 S303F, E452K and L798R cancer type distribution in cBioPortal (https://cbioportal.org) curated non-redundant studies, AACR GENIE (https://genie.cbioportal.org) and COSMIC (https://cancer.sanger.ac.uk) (redundant data removed). **B)** Lollipop diagram of ERBB4 mutations in the cancer types in which S303F, E452K and L798R are most frequently reported in (diagram sourced from AACR GENIE). **C)** The

frequency of co-occurring *ERBB* alterations in patient samples harboring ERBB4 S303F, E452K or L798R mutation, compared to patient samples harboring any ERBB4 alteration. Fisher's exact test was used to compare the ratios. * $P < 0.05$, ** $P < 0.01$, *** $P < 0.001$. Data containing protein coding mutations, copy number alterations and structural variants were sourced from cBioPortal and AACR GENIE (redundant data removed and samples not profiled for all *ERBB* gene alterations excluded). **D)** Co-occurring *ERBB* alterations in cancer samples harboring either *EGFR*, *ERBB2*, *ERBB3*, or *ERBB4* alterations.

Supplementary Table S1. Expression plasmids generated in this work.

**Plasmids generated by Gateway cloning**					
**Plasmid Name**	**Protein change (JM-a CYT-1 numbering)**	**cDNA change (JM-a CYT-1 numbering)**	**Donor vector**	**Destination vector**	**Note**
pBABEpuro-gateway-ERBB4 R106C	R106C	c.316C>T	Commercially ordered from Genewiz	pBABEpuro-gateway (Addgene plasmid #51070; Greulich et al. 2012)	
pBABEpuro-gateway-ERBB4 S303F	S303F	c.908C>T			
pBABEpuro-gateway-ERBB4 R393W	R393W	c.1177C>T			
pBABEpuro-gateway-ERBB4 E452K	E452K	c.1354G>A			
pBABEpuro-gateway-ERBB4 R524C	R524C	c.1570C>T			
pBABEpuro-gateway-ERBB4 R544W	R544W	c.1630C>T			
pBABEpuro-gateway-ERBB4 R711C	R711C	c.2131C>T			
pBABEpuro-gateway-ERBB4 G741E	G741E	c.2222G>A			
pBABEpuro-gateway-ERBB4 S774G	S774G	c.2320A>G			
pBABEpuro-gateway-ERBB4 L798R	L798R	c.2393T>G			
pBABEpuro-gateway-ERBB4 V840I	V840I	c.2518G>A			
pBABEpuro-gateway-ERBB4 R847H	R847H	c.2540G>A			
pBABEpuro-gateway-ERBB4 G870R	G870R	c.2608G>A			
pBABEpuro-gateway-ERBB4 G907E	G907E	c.2720G>A			
pBABEpuro-gateway-ERBB4 R992C	R992C	c.2974C>T			
pBABEpuro-gateway-ERBB4 K1223T	K1223T	c.3668A>C			JM-a CYT-2 numbering: K1207T
pBABEpuro-gateway-ERBB4 S1289A	S1289A	c.3865T>G			JM-a CYT-2 numbering: S1273A
pBABEpuro-gateway-ERBB4 R1304W	R1304W	c.3910C>T			JM-a CYT-2 numbering: R1288W
**Plasmids generated by ligation of oligonucleotides**					
**Plasmid Name**	**Insert**	**Forward oligo**	**Reverse oligo**	**Destination vector**	
Tet-pLKO-neo-shErbb3	TRCN0000023432	CCGGGTTGGATGATTGACGAGAATACTCGAGTATTCTCGTCAATCATCCAACTTTTTG	AATTCAAAAAGTTGGATGATTGACGAGAATACTCGAGTATTCTCGTCAATCATCCAAC	Tet-pLKO-neo (Addgene plasmid #21916; Wiederschain et al. 2009)	

Supplementary Table S2. Patients harboring *ERBB4* alterations and treated with neratinib in the SUMMIT trial. *ERBB4* alteration harboring patients enrolled in PUMA-NER-5201, the SUMMIT trial (NCT01953926), based on a mutation in *ERBB4* (A) or in *ERBB2*(/*ERBB3*) (B) were treated with neratinib as a single agent (240mg/day). Outcomes of neratinib treatment, detailed cancer type and co-altered genes are presented. Patients whose tumors harbored *ERBB4* mutations functionally characterized in this work are indicated with red rectangles. PFS, progression-free survival; OS, overall survival.

A) Qualifying mutation for SUMMIT trial in ERBB4							
**Qualifying mutation**		**Co-altered genes**	**Cancer type**	**Prior lines of therapy**	**Best response**	**PFS (months)**	**OS (months)**
**ERBB4 N465K**		PIK3CA	ovarian clear cell carcinoma	4	PD	1.1	2.2
**ERBB4 R544W**		TP53	rectal adenocarcinoma	6	PD	1.7	14.3
**ERBB4 V840I**		TP53, HER2 amp, CCNE1 amp	peri-ampullary adenocarcinoma	1	PD	1.7	not evaluable (withdrew consent)
**B) Qualifying mutation for SUMMIT trial in another ERBB gene**							
**Qualifying mutation**	**Co-occurring ERBB4 mutation**	**Co-occurring putative drivers**	**Cancer type**		**Best response**	**PFS (months)**	
ERBB2 S310F	ERBB4 Q132*	ERBB2 (D277H), KDM6A, ARID1A, TERT, ERCC4, CDKN2A del, CDKN2B del	bladder urothelial carcinoma		SD	3.5	
ERBB2 S310F	ERBB4 R711C	TP53, CDKN2A, TERT, TGBR1	cutaneous melanoma		SD	1.3	
ERBB2 D769Y	ERBB4 L798R, N138S	ERBB2 amp, TP53, APC, ARID1B, MCL1 amp, MYC amp, CCND3 amp, CCND1 amp, FGF19 amp, FGF4 amp, FGF3 amp, VEGFA amp, RECQL4 del	esophageal adenocarcinoma		PD	1.9	
ERBB2 S310Y	ERBB4 amp	TP53, TERT, ARID1A, EPHA7, RB1, AXL amp, MCL1 amp, AKT2 amp, MYCL amp	bladder/urinary tract cancer		PD	1.2	
